# Evaluating the Influence of Hashimoto's Thyroiditis on Clinico-Pathological Characteristics and Prognostic Outcomes of Middle Eastern Differentiated Thyroid Carcinoma

**DOI:** 10.1155/2024/9929782

**Published:** 2024-09-14

**Authors:** Sandeep Kumar Parvathareddy, Abdul K. Siraj, Nabil Siraj, Saeeda O. Ahmed, Saif S. Al-Sobhi, Fouad Al-Dayel, Khawla S. Al-Kuraya

**Affiliations:** ^1^ Human Cancer Genomic Research Research Center King Faisal Specialist Hospital and Research Centre, Riyadh, Saudi Arabia; ^2^ Department of Surgery King Faisal Specialist Hospital and Research Centre, Riyadh, Saudi Arabia; ^3^ Department of Pathology King Faisal Specialist Hospital and Research Centre, P.O. Box 3354, Riyadh 11211, Saudi Arabia

## Abstract

**Objective:**

Hashimoto's thyroiditis (HT), also known as chronic lymphocytic thyroiditis, represents the most prevalent autoimmune thyroid disorder globally. The potential influence of HT on the clinical and pathological attributes, as well as the clinical outcomes of differentiated thyroid carcinoma (DTC), remains a point of ongoing debate within the medical community. The central focus of this study was to analyze the influence of HT on clinico-pathological characteristics and its prognostic impact in a large cohort of DTC from Middle Eastern ethnicity. *Design, Patients, Measurements*. An extensive analysis involving 1822 DTC patients was conducted to determine the association with clinico-pathological characteristics as well as prognosis, using Chi-square tests and Kaplan-Meier curves.

**Results:**

23.9% (435/1822) of DTC patients were diagnosed with HT. Univariate analysis revealed a positive correlation between presence of HT and clinico-pathological factors such as female gender, younger age, and early stage tumor. In contrast, HT demonstrated a negative association with several aggressive clinical features, including extrathyroidal extension, distant metastasis, recurrent/persistent disease and high-risk categorization by the American Thyroid Association (ATA) guidelines. Despite HT being associated with favorable clinico-pathological features in Middle Eastern DTC patient, our study found no significant influence on overall survival or recurrence-free survival.

**Conclusion:**

The finding of an association between HT and favorable clinico-pathological characteristics, but lack of impact on prognosis, underscores the complexity of HT-DTC relationship, necessitating further comprehensive research to fully understand these interactions.

## 1. Introduction

Hashimoto's thyroiditis (HT), also known as chronic lymphocytic thyroiditis, is the most prevalent autoimmune disorder characterized by chronic inflammation of thyroid gland [1]. This condition is a significant contributor to the global incidence of hypothyroidism and causes a wide range of thyroid metabolic and physiological disturbances [2, 3]. The worldwide prevalence of HT exhibits marked geographical variability, attributed to genetic, environmental, and lifestyle factors. Intriguingly, the prevalence remains high within iodine-sufficient populations, hinting at the complexity of factors influencing the onset and progression of this autoimmune disorder [4]. In addition, differentiated thyroid carcinoma (DTC) accounts for a considerable proportion of thyroid malignancies, with its incidence rate steadily increasing globally [5–7]. The rise in DTC incidence has been associated with improved diagnostic techniques, environmental factors and possibly an actual increase in disease occurrence [6, 8].

Coexistence of HT with DTC was observed for a very long time [9]. Ever since, the potential interplay between HT and DTC, particularly regarding the influence of HT on clinico-pathological features and prognosis of DTC has been a topic of ongoing debate within the medical research community. The current literature offers dichotomous perspective, with a subset suggesting significant impact of HT on DTC progression and others finding no such association [10–16]. This divergence in scientific opinion is further emphasized within the Middle Eastern ethnicity, where investigation of the relationship between HT and DTC is relatively sparse [17, 18]. Given the unique genetic and environmental context of this population, exploring this association within a large cohort of Middle Eastern DTC is crucial to enhancing our understanding of HT-DTC interactions.

Therefore, our study aims to elucidate the prevalence of HT among DTC patients and unravel the potential impact of HT on clinico-pathological features and prognosis of DTC among Middle Eastern individuals.

## 2. Materials and Methods

### 2.1. Clinical Cohort

One thousand eight hundred and twenty-two DTC patients diagnosed between 1988 and 2018 at King Faisal Specialist Hospital and Research Centre (Riyadh, Saudi Arabia) were included in the study. The Institutional Review Board of the hospital approved this study and since only retrospective patient data were used, the Research Advisory Council (RAC) provided waiver of consent under project RAC # 221 1168 and # 2110 031. The study was conducted in accordance with the Declaration of Helsinki.

### 2.2. Clinico-Pathological and Follow-Up Data

Baseline clinico-pathological data were collected from case records and have been summarized in Table 1. Staging of DTC was performed using the eighth edition of American Joint Committee on *Cancer* (AJCC) staging system [19]. Patients were stratified into low, intermediate and high risk based on 2015 American Thyroid Association (ATA) guidelines [20]. Patient follow-up and definition of persistent/recurrent disease has been described previously by us [21–24]. Briefly, low-risk DTC patients were followed up annually, intermediate risk patients were followed up at 6 months' intervals and high risk patients were followed up at 3 months' intervals. At each follow-up, neck ultrasound, thyroid function tests, thyroglobulin (Tg) levels and thyroglobulin antibodies were performed. FT4, TSH, Tg and anti-Tg antibody levels were measured using commercially available competitive electrochemiluminescence immunoassay (Roche Elecsys). In addition, for high risk patients, whole body scan (WBS) and/or PET CT scan were performed to identify tumor recurrence. Presence of active disease within 12 months post-operatively was defined as persistent disease, whereas patients who had excellent response for at least 12 months post-operatively and later developed active disease were defined as having recurrent disease.

The diagnosis of HT was based on histopathological finding of lymphoplasmacytic infiltration with germinal center and the presence of large follicular cells with abundant granular eosinophilic cytoplasm.

The study endpoint for our analysis was overall survival (OS) and recurrence-free survival (RFS). OS was defined as the time (in months) from date of initial surgery to the date of death due to any cause. RFS was defined as the time (in months) from date of initial surgery to the date of development of recurrent disease.

### 2.3. Statistical Analysis

The associations between clinico-pathological variables was performed using contingency table analysis and Chi-square tests. Survival curves were generated using the Kaplan-Meier method. Mantel-Cox log-rank test was used to evaluate significance. Two-sided tests were used for statistical analyses with a limit of significance defined as *p* value < 0.05. Data analyses was performed using the JMP14.0 (SAS Institute, Inc., Cary, NC) software package.

## 3. Results

### 3.1. Patient Characteristics

Median age of the study population was 38.9 years (range: 6.0 – 87.0 years), with a male: female ratio of 1 : 3. 31.3% (567/1822) of tumors were bilateral and 48.4% (877/1822) were multifocal. Extrathyroidal extension was noted in 39.9% (727/1822) of DTCs. Regional lymph node metastasis (LNM) was noted in 53.1% (846/1594, with 228 cases being classified as Nx) of cases and distant metastasis was present in 9.2% (167/1822) (Table 1).

### 3.2. Hashimoto's Thyroiditis in Differentiated Thyroid Cancer and Its Clinico-Pathological Associations

HT was noted in 23.9% (435/1822) of DTC patients. HT in DTC patients was significantly associated with younger age (<55 years; *p*=0.0209), female gender (*p*=0.0447) and stage I tumors (*p*=0.0036). In addition, HT was inversely associated with extrathyroidal extension (*p*=0.0372), distant metastasis (*p*=0.0003), ATA high risk (*p*=0.0041) and recurrent/persistent disease (*p*=0.0155) (Table 1). However, no association was found with tumor size, laterality, focality, lymphovascular invasion and lymph node metastasis.

**Figure 1:**
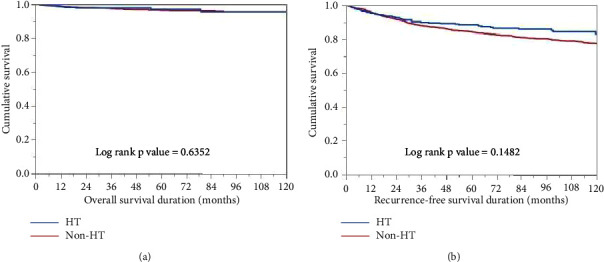
Hashimoto's thyroiditis (HT) and prognosis in differentiated thyroid carcinoma. Kaplan-Meier survival plot showing no statistically significant difference between HT and non-HT DTC patients with respect to (a) overall survival and (b) recurrence-free survival.

We next sought to determine the role of HT in prognosis of DTC patients. However, no significant difference was found between HT and non-HT patients with respect to OS (*p*=0.6352) and RFS (*p*=0.1482) (Figures 1(a) and 1(b)).

## 4. Discussion

The findings from this study provide insights into the hypothesized association between HT and DTC. Out of 1822 DTC patients analyzed, 23.9% had concurrent HT, a finding that aligns with global reports of HT prevalence among DTC patients [25–27]. This co-occurrence highlights the potential interplay between autoimmune process and thyroid tumorigenesis, a subject of ongoing research [28, 29].

Our analysis of clinico-pathological characteristics revealed a direct correlation between HT and certain demographic and disease related variables, including female gender, younger age and early tumor stage. These observations are consistent with previous studies that have reported higher incidence of HT among females and younger individuals, possibly due to hormonal and immunological differences [30–34]. Furthermore, our finding of an association with early tumor stage bolsters the hypothesis that autoimmune response inherent in HT could potentially modulate DTC progression, resulting in diagnosis at earlier stage [32, 35, 36]. Interestingly, our study found an inverse relationship between HT and aggressive clinico-pathological features of DTC, such as extrathyroidal extension, distant metastasis, recurrent/persistent disease, and high ATA risk. This observation is in concordance with prior studies that reported less aggressive clinical features of DTC in patients with concurrent HT [31, 34, 37, 38].

However, despite aforementioned correlations, we found that HT did not significantly influence the overall prognosis of Middle Eastern DTC patients. There was no discernible association with overall survival or recurrence-free survival, two pivotal indicators in the prognostic assessment of DTC. This finding is in contrast to some prior research, which suggested a protective role of HT in DTC prognosis [37, 39–41]. However, our findings align with other studies that reported no significant impact of HT on DTC outcome [11, 42, 43]. This discrepancy highlights the complexity of HT-DTC interaction and underscores the necessity for more comprehensive investigations.

Several studies with different cohort sizes and ethnic background have attempted to unravel this intricate relationship with variable results. For instance, a study by Lun et al. [32] suggested that the presence of HT was associated with less aggressive clinical features and better prognosis. Similarly, Liang et al. [40] and Ryu and Yoon et al. [41] reported a higher disease-free survival and recurrence-free survival, respectively, in PTC patients with HT compared to those without HT. In contrast, Lee et al. [11] found no significant difference in the prognosis of DTC patients with or without HT. These contrasting results underline the heterogeneity of findings in this subject. Moreover, Dobrinja et al. [42] reported an association between the presence of HT and less aggressive features but found no impact on recurrence or survival rate, thereby introducing another facet to our understanding of the HT-DTC relationship. Interestingly, recent metaanalyses by Moon et al. [39] and Lee et al. [15] concluded that HT was associated with favorable prognosis in patients with PTC, primarily through an association with less aggressive tumor characteristics. Another study by Jeong et al. [37] showed that co-existence of HT and DTC was associated with higher disease-free survival rate on univariate analysis, but was not significant on multivariate analysis. These findings, in conjunction with ours, underline the existing uncertainty regarding the prognostic role of HT in DTC and the need for a more nuanced understanding of HT-DTC interaction.

## 5. Conclusions

In summary, our study shows that, while HT may influence certain clinico-pathological features in DTC, it does not appear to significantly impact prognosis of DTC patients from Middle Eastern ethnicity. This observation reinforces the need for future research incorporating larger cohorts and diverse ethnic groups to understand the multifaceted relationship between HT and DTC.

## Figures and Tables

**Table 1:** Clinical and histopathological associations of patients with and without Hashimoto's thyroiditis (HT).

Parameters	All patients		HT		Non-HT		*p* value	Odds ratio (95% CI)
	No	%	No	%	No	%		
Total	1822		435	23.9	1387	76.1		
Age (years)								
Median (range)	38.9 (6–87)		38.0 (9–87)		39.0 (6–87)		0.0615	
<55	1472	80.8	368	84.6	1104	79.6	0.0209	
≥55	350	19.2	67	15.4	283	20.4		1.41 (1.05–1.88)
Gender								
Female	1375	75.5	344	79.1	1031	74.3	0.0447	1.31 (1.01–1.69)
Male	447	24.5	91	20.9	356	25.7		
Tumor histotype								
PTC	1716	94.2	410	94.2	1306	94.2	0.9425	1.02 (0.64–1.61)
FTC	106	5.8	25	5.8	81	5.8		
Variants of PTC								
Classical variant	1080	62.9	244	59.5	836	64.0	0.3897	Reference
Follicular variant	301	17.6	77	18.8	224	17.1		1.18 (0.88–1.58)
Tall cell variant	180	10.5	46	11.2	134	10.3		1.18 (0.82–1.69)
Other variants	155	9.0	43	10.5	112	8.6		1.32 (0.90–1.92)
Tumor size (mm) mean ± SD	29.6 ± 19.5		28.6 ± 18.2		29.9 ± 19.9		0.2254	
Tumor laterality								
Unilateral	1247	68.7	296	68.0	951	69.0	0.7190	
Bilateral	567	31.3	139	32.0	428	31.0		1.04 (0.83–1.32)
Multifocality								
Yes	877	48.4	210	48.3	667	48.4	0.9731	0.99 (0.80–1.24)
No	937	51.6	225	51.7	712	51.6		
Extrathyroidal extension								
Present	727	39.9	155	35.6	572	41.2	0.0372	0.79 (0.63–0.98)
Absent	1095	60.1	280	64.4	815	58.8		
Lymphovascular invasion								
Present	524	28.8	136	31.3	388	28.0	0.1859	1.17 (0.93–1.48)
Absent	1298	71.2	299	68.7	999	72.0		
Lymph node metastasis								
Present	846	53.1	191	51.8	655	53.4	0.5644	0.93 (0.74–1.18)
Absent	748	46.9	178	48.2	570	46.5		
Distant metastasis								
Present	167	9.2	21	4.8	146	10.5	0.0003	0.43 (0.27–0.69)
Absent	1655	90.8	414	95.2	1241	89.5		
TNM stage								
I	1531	84.4	388	89.4	1143	82.8	0.0036	Reference
II	198	10.9	27	6.2	171	12.4		0.47 (0.31–0.71)
III	24	1.3	6	1.4	18	1.3		0.98 (0.39–2.49)
IV	62	3.4	13	3.0	49	3.5		0.78 (0.42–1.46)
ATA risk category								
Low	339	18.6	117	26.9	222	16.0	0.0041	Reference
Intermediate	635	34.9	165	37.9	470	33.9		1.10 (0.82–1.49)
High	848	46.5	153	35.2	695	50.1		0.74 (0.55–0.99)
Recurrent/persistent disease								
Present	602	33.0	123	28.3	479	34.5	0.0155	0.68 (0.50–0.92)
Absent	1220	67.0	312	71.7	908	65.5		
Anti-Tg antibody mean (±S.D.)	160.6 ± 547.94		153.7 ± 498.5		163.3 ± 565.8		0.7712	

## Data Availability

The data that support the findings of this study are available from the corresponding author upon reasonable request.
